# Effects of Carbon Ion Beam Irradiation on Butanol Tolerance and Production of *Clostridium acetobutylicum*

**DOI:** 10.3389/fmicb.2020.602774

**Published:** 2020-12-18

**Authors:** Yue Gao, Miaomiao Zhang, Xiang Zhou, Xiaopeng Guo, Cairong Lei, Wenjian Li, Dong Lu

**Affiliations:** ^1^Institute of Modern Physics, Chinese Academy of Sciences, Lanzhou, China; ^2^Chinese Academy of Sciences, University of Chinese Academy of Sciences, Beijing, China; ^3^Gansu Key Laboratory of Microbial Resources Exploitation and Application, Lanzhou, China

**Keywords:** *Clostridium acetobutylicum*, carbon ion beam irradiation, mutant, membrane permeability, response surface methodology

## Abstract

*Clostridium acetobutylicum* (*C. acetobutylicum*) has considerable potential for use in bioenergy development. Owing to the repeated use of traditional mutagenesis methods, the strains have developed a certain tolerance. The rheology of the bioprocess and the downstream processing of the product heavily depend on the ability of *C. acetobutylicum* mutants to produce butanol. Carbon ion beam irradiation has advantages over traditional mutation methods for fermentative production because of its dose conformity and superb biological effectiveness. However, its effects on the specific productivity of the strains have not been clearly understood. In this study, we screened five mutants through carbon ion beam irradiation; mutant Y217 achieved a butanol-production level of 13.67 g/L, exceeding that of wild-type strain ATCC 824 (i.e., 9.77 g/L). In addition, we found that the mutant maintained normal cell membrane integrity under the stimulation of 15 g/L butanol, whereas the intracellular macromolecules of wild-type strain ATCC 824 leaked significantly. Subsequently, we used the response surface methodology (RSM) to determine if the mutant cell membrane integrity improved the butanol tolerance. We verified that with the addition of butanol, the mutant could be fermented to produce 8.35 g/L butanol, and the final butanol concentration in the fermentation broth could reach 16.15 g/L. In this study, we proved that under butanol stress, mutant Y217 features excellent butanol production and tolerance and cell membrane integrity and permeability; no prior studies have attempted to do so. This will serve as an interesting and important illustration of the complexity of genetic control of the irradiation mutation of *C. acetobutylicum* strains. It may also prove to be useful in the bioengineering of strains of the mutant for use in the predevelopment stage.

## Introduction

The exhaustive exploitation of fossil fuels has led to a decline in the storage of fossil energy worldwide. Under the multiple pressures of energy depletion and environmental degradation, the development of alternative clean energy is urgently needed (Jiaquiang et al., 2018; Zhang et al., 2019). Presently, the main widely used alternative biofuels include biomethanol, biodiesel, bioethanol, and biobutanol. However, biobutanol significantly differs from other biofuels. As an essential product of microbial fermentation, biobutanol has the advantages of low volatility and high energy, and it can be mixed with gasoline in any ratio. Thus, it has significant potential for use in bioenergy development (Kikuchi et al., 2009). However, cost and efficiency are factors that limit the production of butanol by *Clostridium acetobutylicum*. Presently, acetone-butanol-ethanol (ABE) fermentation predominantly uses food crops (corn) and non-food crops (straw) as raw materials, which are expensive and have high pretreatment costs (Kumar et al., 2012). During the ABE fermentation process, when the concentration of butanol in the fermentation product exceeds 13 g/L, it has a toxic effect on *Clostridia*; the thallus begins to autolyzed, or spores are formed, and the fermentation process is gradually terminated, resulting in low butanol production and conversion rate, which limits the industrial production (Dadgar and Foutch, 1988). Therefore, rapid and efficient strain-modification techniques can improve the metabolic capacity and enhance the ABE production efficiency.

Mutagenesis is a reliable technology and widely used in strain improvement. Mutagenesis methods, including chemical and physical mutagenesis methods, are commonly used in mutagenic engineering to activate *Clostridia* strains. The strains are then screened by a selective medium to obtain mutants with excellent performance and applicability. Zhang et al. (1996) used N-methyl-N-nitro-N-nitrosoguanidine (NTG) and ethyl methane sulfonate (EMS) to obtain mutants with a high butanol ratio by performing chemical mutagenesis on *C. acetobutylicum*. The proportion of butanol in the total solvent is stable at approximately 70%, which is approximately 10% higher than that of the wild-type strain. Li et al. (2013) used *C. beijerinckii* as a starting strain and combined the low-energy ion beam implantation technology, NTG induction, and rational screening model to modify the strain; finally, they obtained a mutant with excellent cell performance and significantly increased the butanol production. The butanol yield of MUT3 reached 15.8 ± 0.7 g/L, which was 1.46 times higher than that of the starting strain in the P2 medium.

Owing to the repeated use of traditional mutagenesis methods, some industrial strains have developed a certain tolerance. Therefore, researchers have begun to develop new methods for microbial mutagenesis breeding; heavy ion beam irradiation, as a more efficient irradiation method, has been extended to a variety of scientific research and biological mutagenesis breeding, achieving a remarkable effect with high economic benefit. The metabolites of gentamicin-producing bacteria and respiration-deficient mutants were increased by heavy ion beam mutagenesis (Xie et al., 1995; Guo et al., 2019). In the food industry, new strains with a high conversion rate have also been obtained by heavy ion beam irradiation and industrialized; these include *Aspergillus Niger* and *Saccharomyces cerevisiae*, which have been screened for stable high-yield strains (Yan et al., 2009; Hu and Chen, 2012). In terms of microbial energy, the use of heavy ion beam irradiation produced poly-β-hydroxybutyrate (PHB) high-yield strain G15 (Xue et al., 2010) and oil-producing microalgae mutant strain D90G-19. Cell membranes provide an effective barrier to environment; therefore, adjusting the membrane permeability involves the regulation of passage of ions, nutrients, and toxic substances through the membrane (Qi et al., 2019). Related studies have been conducted to improve the solvent production by changing the cell membrane permeability of *C. acetobutylicum.* For example, Xin et al. (2018) reported that after supplementing 15% Tween 80 (v/v), the butanol production increased to 18.65 g/L, which is 38% higher than that for the control group, and the butanol tolerance increased to 18 g/L, which is 80% higher than that for the control group. Dhamole et al. (2012) conducted a series of tests with non-ionic surfactants (Triton X-114, L64, L62LF, L61, and L62) to increase the production of acetone and butanol. The results showed that L62 not only significantly improved the butanol yield but also produced a better butanol extractant. In addition, changes in cell membrane permeability are frequently identified as one of the main effects of butanol stress-induced toxicity (Qi et al., 2019).

The traditional exogenous butanol method, one-factor-at-a-time approach, is not feasible for establishing relationships between all the experimental input factors and the output response. Although the traditional approach can be useful to find the predominant factors, it consumes considerable time and energy. Furthermore, because the results are valid only under fixed experimental conditions, the predictions for other conditions may be inaccurate. To solve this problem, the design of experiment (DOE) offers a better alternative for studying the effects of butanol tolerance of *C. acetobutylicum* and their response with a minimum number of experiments. The response surface methodology (RSM) is a multifactor, multilevel test-design method that uses multiple quadratic regression equations to fit the functional relationship between factors and response values. It is typically used to obtain the best process parameters by analyzing the regression equations, which is a statistical method to solve multivariable problems (Ebrahimi et al., 2020; Figueroa-Torres et al., 2020). Liu et al. (2010) used the RSM to optimize the medium for xylose fermentation for producing butanol and established a regression equation. This optimized medium was obtained and the yield of butanol increased by 18.4%, reaching 6.69 g/L under optimized condition. Using the RSM-based DOE, the aggregate mix proportions can be obtained with a minimum number of experiments without the need for performing all the possible combination experiments. Further, the input levels of the different variables for a particular level of response can be determined. The RSM is a collection of statistical techniques for designing experiments, building models, evaluating the effects of factors, and searching for the optimum conditions of the factors. It also quantifies relationships between one or more measured responses and the vital input factors.

In this study, we used the heavy ion beam irradiation mutagenesis method for the selection of *C. acetobutylicum* to obtain a new strain with potential fermentation performance, attempt to use it in the studies of membrane permeability of the *C. acetobutylicum* mutant and responses of exogenous butanol with minimum numbers of experimental runs, determine the optimum tolerance using the RSM, and analyze the relationship between butanol tolerance and production of mutant. Improving butanol tolerance is critical for future applications of this process in the fermentation industry.

## Materials and Methods

### Irradiation Treatment and Screening Process

*Clostridium acetobutylicum* strain ATCC 824 was preserved in 40% glycerol at –80°C. The growth medium of *C. acetobutylicum* strains was the liquid reinforced *Clostridial* medium (RCM) with 0.5% glucose or RCM agar plate at 37°C under anaerobic conditions. The following irradiation treatment was performed: wild-type strain *C. acetobutylicum* ATCC 824 was cultured on the RCM liquid medium at 37°C for 20 h (OD_600_ = 0.8–1.0); then, 1 ml of bacterial suspension was transferred onto a 35-mm irradiation dish. Six doses of cell suspension were irradiated at 30, 60, 90, 120, 150, and 200 Gy, and three samples were treated with every dose. Irradiation mutagenesis tests were conducted by the Heavy-Ion Research Facility in Lanzhou (HIRFL), Institute of Modern Physics, Chinese Academy of Sciences, with 80 MeV/u carbon ion beams. The survival fractions were measured by normalizing the colony counts of irradiated cells with those of untreated cells; survival fraction = (average number of colonies on treatment plates/average number of colonies on untreated plates) × 100%. The following screening process was performed: cell suspensions were diluted to 10^–4^, and 200 μL of the dilution was spread on the RCM agar plate and cultured at 37°C for 36–38 h. Mutant strains were screened through the detection of starch utilization and butanol tolerance concentration. The screening agar plate, which contained 2.0% (v/v) butanol and 0.2% starch, was poured onto the RCM plate. The positive mutants were primarily selected by recording and comparing the diameter sizes of the transparent circles. The composition of RCM in distilled water was as follows: beef extract, 10.00 g/L; peptone, 10.00 g/L; yeast extract, 3.00 g/L; glucose, 5.00 g/L; starch, 1.00 g/L; NaCl, 5.00 g/L; sodium acetate, 3.00 g/L; cysteine hydrochloride, 0.50 g/L; (agar plate, 15 g/L).

### Fermentation Conditions

The batch fermentation study was conducted in a 250 ml screw-capped bottle with 50 ml of corn- powder medium (CM). The spore suspension of *C. acetobutylicum* ATCC 824 or its mutant strains was anaerobically cultured to the stationary phase at 37°C in the RCM. The screw-capped bottles were inoculated with 5–10% seed culture of the total fermentation culture volume and the stationary culture at 37°C. The samples were drawn periodically to monitor the pH values and concentrations of the solvents and acids. The fermentation studies were performed in triplicate. The CM was used for the main batch fermentation. To prepare the CM, 70 g of corn powder was suspended in 1 L of distilled water and boiled for 1 h; the composition of CM in distilled water was as follows: 70 g/L corn powder, 3.0 g/L (NH4)_2_SO_4_, 3.0 g/L CaCO_3_, 1.0 g/L K_2_HPO_4_⋅3H_2_O, 1.25 g/L KH_2_PO_4_, 0.1 g/L MgSO_4_⋅7H_2_O, and 0.01 g/L FeSO_4_⋅7H_2_O. All the purchased chemicals were >99% pure. To maintain complete anaerobic conditions, all the media were purged with N_2_ to remove O_2_, and agar plates were cultured in anaerobic jars.

### Determination of the Genetic Stability of Mutants

The growth abilities and solvent yields were determined for 1–12 generations of the mutant and wild-type strain. A generation was defined as follows: a single colony was picked and inoculated into a 250 ml screw-capped bottle with 50 mL of the CM from the RCM plate and static culture at 37°C until OD_600_ approaches 1.0, after which the diluted suspension was plated on the RCM plate. The same procedure was followed for each generation. The growth rate was expressed by biomass concentration (dry cell weight, DCW). A 15 mL centrifuge tube was placed in an oven at 60°C for drying. The empty centrifuge tube was accurately weighted to X, 10 mL of fermentation broth was drawn into the centrifuge tube and centrifuged at 4000 × *g* for 15 min. After the supernatant was discarded, the bacterial pellet was washed five times with distilled water, dried in an oven at 60°C to a constant weight, and weighed to Y. DCW (g/L) = (Y–X)/10 × 1000. Fermentation experiments were performed as detailed previously. The butanol yield and total solvent content were detected.

### Scanning Electron Microscopy (SEM) Sample Preparation

The suspension of the late logarithmic growth period liquid medium was collected by centrifugation at 8000 × *g* for 3–5 min and washed twice with a phosphate buffer (pH = 7.2) used for SEM observation. Thereafter, 2.5% glutaraldehyde was added to immobilize the cells at 4°C for 2–3 h. The cells were centrifuged (8000 × *g* for 3 min) and washed twice with a phosphate buffer (pH = 7.2), and a series of 10–100% ethanol was used for gradient dehydration. Each concentration was maintained for 15 min, and 100% dehydration was performed twice. Finally, the specimens were dried at room temperature (25°C) and sprayed with gold. The samples were used for SEM observation (FEI Nova NanoSem 450, working voltage: 15 kV, amplification factor: 80,000).

### Detection of Intracellular Protein and Nucleotide Diffusion

After the sample was treated with 15 g/L butanol, the cells were collected by centrifugation (8000 × *g*, 4°C, 10 min) and washed and suspended in a phosphate buffer (pH = 7.2) to achieve a final concentration of 10^6^–10^7^ cells/mL. To ensure enzyme activity, all the samples were placed in an ice water bath for 2 h and then centrifuged (8000 × *g*, 4°C, 10 min) to collect the cells. The bicinchoninic acid (BCA) kit, Biyuntian, was used for protein determination. Following the BCA instruction, the optical density (OD) of experimental group and Bovine Serum Albumin (BSA) standard group (0–200 μg/mL) were measured every 2 h at 560 nm after 37 h of incubation. The BSA standard group was used to draw the standard curve. In addition, the intracellular nucleotides were measured using the following formula: Nucleotide (μg/mL) = (11.87 × OD_260_ –10.40 × OD_280_) × 100/9, where OD_260_ and OD_280_ are the optical density of the bacterial suspension at 260 and 280 nm, respectively.

### Experimental Design and Validation by RSM

Based on the Box-Behnken design, the Design-Expert 8.0.6 software was used to analyze and select butanol concentration (*X*_1_), temperature (*X*_2_), and pH (*X*_3_) as the investigation factors. The butanol production (*Y*) was the response value, and the RSM was used to evaluate the effects of three important factors on butanol fermentation and butanol tolerance of the carbon ion beam-irradiated mutant. The effect of each variable was studied at three different coded levels (–1, 0, +1) with low, medium, and high values (Table 1). The RSM analysis regression model is given by

**TABLE 1:** Actual values and level values of the variables employed in RSM.

**Variables**	**Range and level**		
	**−1**	**0**	**+1**
*X*_1:_ Butanol(g/L)	6	8	10
*X*_2:_ Temperature(°C)	34	36.5	39
*X*_3:_ pH	6.2	6.8	7.4

$$Y=\beta_{0}+\sum_{i=1}^{3}\beta_{i}\,X_{i}+\sum_{i=1}^{3}\beta_{i\,i}\,X_{i}^{2}+\sum_{i<j=2}^{3}\beta_{i\,j}\,X_{i}\,X_{j},$$

where Y is the predicted response, β_*0*_ the offset term,β_*i*_ the linear coefficient,β_*ii*_ the squared coefficient,β_*ij*_ the interaction coefficient, and *X*_*i*_ and *X*_*j*_ are the independent variables.

### Analytical Methods

The pH of the fermentation broth was measured using a Mettler Toledo pH meter (Giesen, Germany). Before the analysis of the product concentration, the fermentation broth was analyzed by centrifugation at 8000 *g* for 5 min, and the supernatant was filtered using a 0.22 μm syringe filter. Solvents (acetone, ethanol, and butanol) and acids (acetic acid and butyric acid) were determined by 456-GC gas chromatography (Bruker, Germany) on a HP-INNOWAX column (30 m × 0.320 mm × 0.50 μm; Agilent J&W, United States) equipped with a flame ionization detector.

### Statistical Analysis

All experiments were performed at least three times and all the data were reported as mean ± SD. Statistical analysis software (Microsoft Excel 2016) was used to analyze the statistical significance of the experimental data, and *p* < 0.05 indicated a significant difference and *p* < 0.01 indicated a highly significant difference. RSM experiments were analyzed by ANOVA.

## Results

### Survival and Mutagenesis of *C. acetobutylicum* Irradiated With the Carbon Ion Beam

**FIGURE 1:**
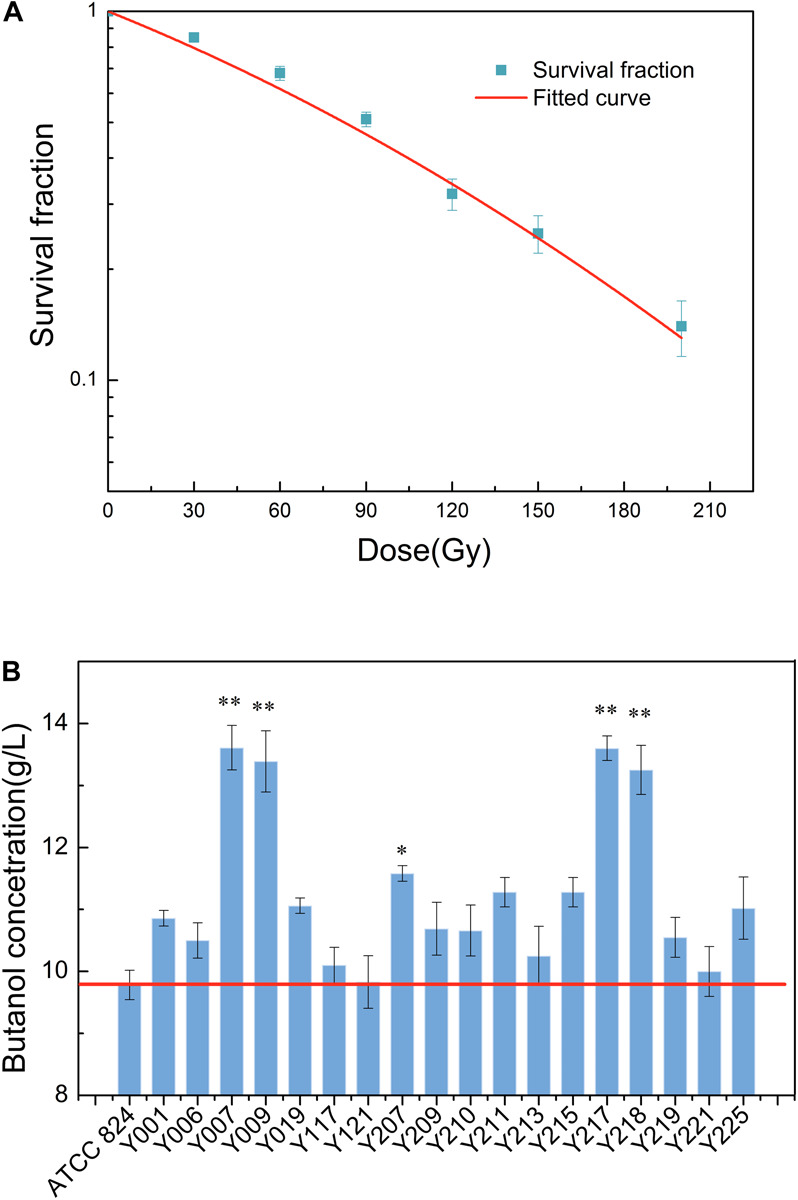
**(A)** Survival fraction of *C. acetobutylicum* ATCC 824 treated with different doses (30, 60, 90, 120, 150, and 200 Gy) of carbon ion beam irradiation. **(B)** Carbon ion beam irradiation screening for high butanol-production mutants. Data of the total butanol concentrations of the mutants in 250 mL screw-capped bottles after carbon ion beam irradiation mutagenesis are presented. Y001–Y225 denote the mutants. *p < 0.05, **p < 0.01.

This test successfully applied carbon ion beam irradiation to *C. acetobutylicum*. Due to a lack of reference conditions for the carbon ion beam irradiation of *C. acetobutylicum*, we investigated the survival fractions using six doses of carbon ion beam irradiation in this test, based on reference doses for other microbial strains irradiated with the carbon ion beam. Survival fraction reflects the damage degree to organisms caused by external stimuli. Studying the survival fraction of *C. acetobutylicum* cells after heavy ion beam irradiation can be used to evaluate the damage degree of *C. acetobutylicum* cells from heavy ion beams. It is also used to determine the optimal irradiation dose. The survival curve of *C. acetobutylicum* ATCC 824 cells after carbon ion beam irradiation was measured using a plate colony counting method. Figure 1A shows that at 30, 60, 90, 120, 150, and 200 Gy, the survival fractions were 0.85, 0.68, 0.51, 0.32, 0.25, and 0.14, respectively. With the increase in the irradiation dose, the survival fraction showed a significant downward trend. The 50% lethal dosage was 90 Gy and after 200 Gy irradiation, the cell survival fraction reduced to 14%. In this study, mutants were obtained using the higher carbon ion irradiation dose (120 Gy), causing a higher cell mortality (approaching 70%).

After two rounds of selective plate primary screening and secondary fermentation screening, five mutants with higher biobutanol accumulation were screened and selected. In comparison with the wild-type strain, butanol production by the screened strains Y007, Y009, Y207, Y217, and Y218 was greatly improved in terms of the final butanol concentration (Figure 1B). The wild-type strain and the five mutants, Y007, Y009, Y207, Y217, and Y218 could synthesize (9.78 ± 0.23), (13.61 ± 0.35), (13.39 ± 0.49), (11.58 ± 0.12), (13.60 ± 0.19), and (13.25 ± 0.39) g/L butanol, respectively. The increase in butanol production was considerable for the Y007, Y009, Y217, and Y218 strains, which showed the greatest production ability with an increase of 35–39% in butanol production. These results show that carbon ion beam irradiation can be applied as a useful candidate tool in *C. acetobutylicum* breeding.

### Fermentation Kinetic Characteristics of the Wild-Type Strain and Mutants

**FIGURE 2:**
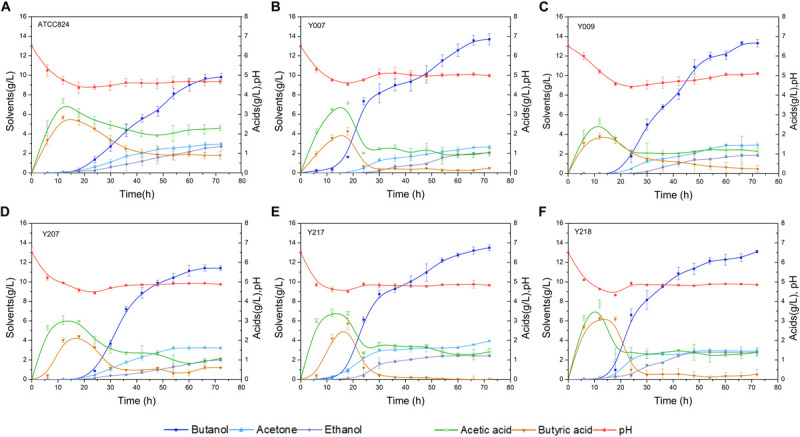
Batch fermentation kinetics of wild-type strain and five mutants in 250 mL screw-capped bottles with an initial corn powder concentration of 70 g/L. **(A)** *C. acetobutylicum* ATCC 824. **(B)** Y007, **(C)** Y009, **(D)** Y207, **(E)** Y217, and **(F)** Y218. The data are the averages of three replicated experiments, and the error bar represents the standard deviation.

The screw-capped bottle fermentation characteristic curves for the wild-type and five mutant strains are shown in Figure 2. There is a significant difference in final product concentrations; the five mutant strains produced higher butanol concentrations than the wild-type strain (9.81 g/L butanol). Y007, Y009, Y207, Y217, and Y218 produced 13.69, 13.30, 11.40, 13.50, and 13.10 g/L of butanol, respectively. Among them, mutant Y009 and Y207 reached the highest concentrations in 66 h, i.e., 6 h before the other strains (Figures 2C,D). The butanol productivity of Y009 reached 0.2 g/L/h, which indicates a 47.79% improvement over that of the wild-type strain. Correspondingly, the total solvents production of the five mutant strains was 18.38, 18.03, 16.59, 19.86, and 18.77 g/L, respectively, each of which was above the wild-type (15.46 g/L). Acid production by all strains showed a similar tendency, namely that it increased significantly before 20 h and then decreased gradually. The total acid (acetic acid and butyric acid) concentrations in cultures of the five mutant strains were 1.29, 1.35, 1.66, 1.41, and 1.63 g/L, respectively, each lower than that of the wild-type strain (3.20 g/L). Compared to the wild-type strain, butanol in Y007 and Y009 constituted over 70% of the total solvents. Strain Y217 (The raw sequence data of Y217 have been deposited in the Genome Sequence Archive in National Genomics Data Center, Beijing Institute of Genomics, Chinese Academy of Sciences, under accession number CRA003426, see Supplementary Material for details), with high butanol and solvent characteristics, selected from the 120 Gy irritated bacterial suspension, was chosen to be the experimental subject.

### Discrepant Genetic Stability of Mutants

**FIGURE 3:**
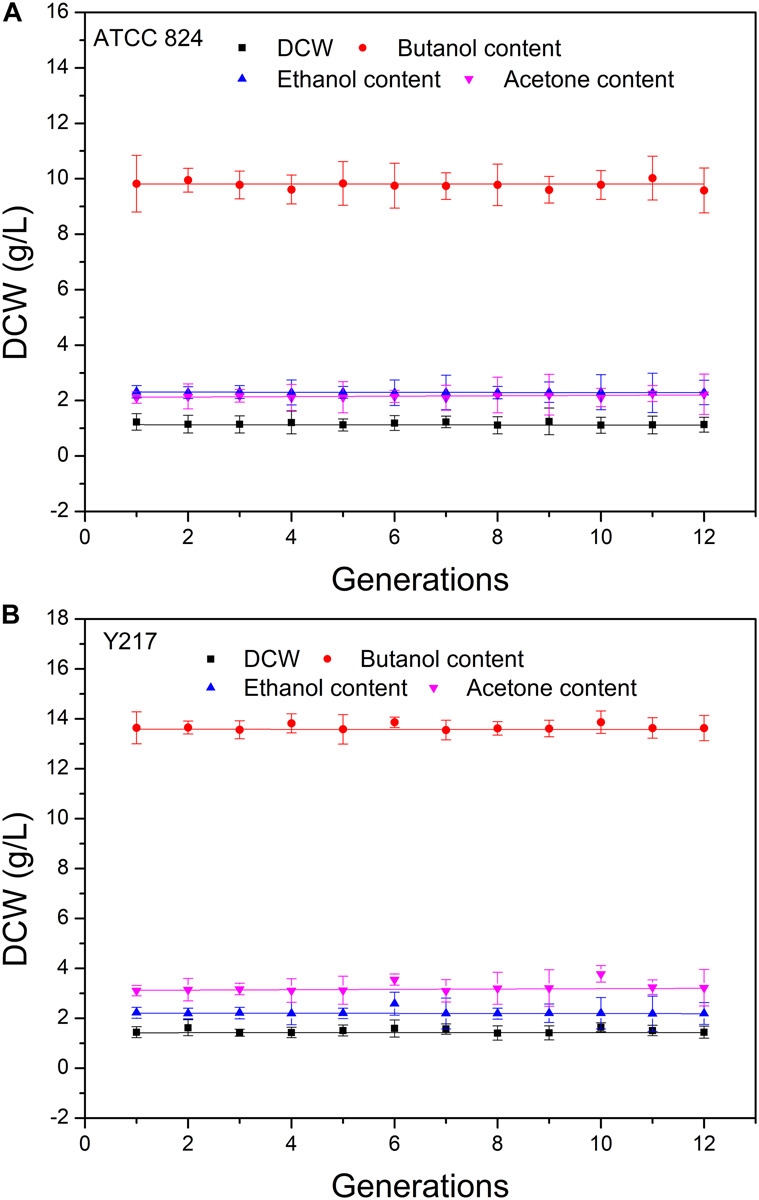
Stability of mutant and the wild-type strain cell dry weight (DCW), butanol content, ethanol content, and acetone content were evaluated for 1–12 generations. **(A)** *C. acetobutylicum* ATCC 824. **(B)** Y217. The detected values of the indexes and generations were linearly fitted with slopes approaching 0.

We determined the genetic stability of the mutant using consecutive generation experiments. We evaluated the genetic stability of the mutant by measuring the growth intensity and the butanol, ethanol, and acetone contents for 1–12 generations. For each strain, the change of measured value of each investigation index by generation was well linearly fitted, and the slope of the fitted straight line approached 0, which indicated that the measured indexes were very stable within the 12th generation of the mutant (Figure 3). In addition, the growth ability and fermentation performance of the mutant were significantly higher than the wild-type strain. These results indicate that the carbon ion beam mutagenesis technique can generate genetically stable microbial strains with strong product metabolite accumulation ability.

### Intracellular Protein and Nucleotide Diffusion of *C. acetobutylicum* Under Butanol Stress

**FIGURE 4:**
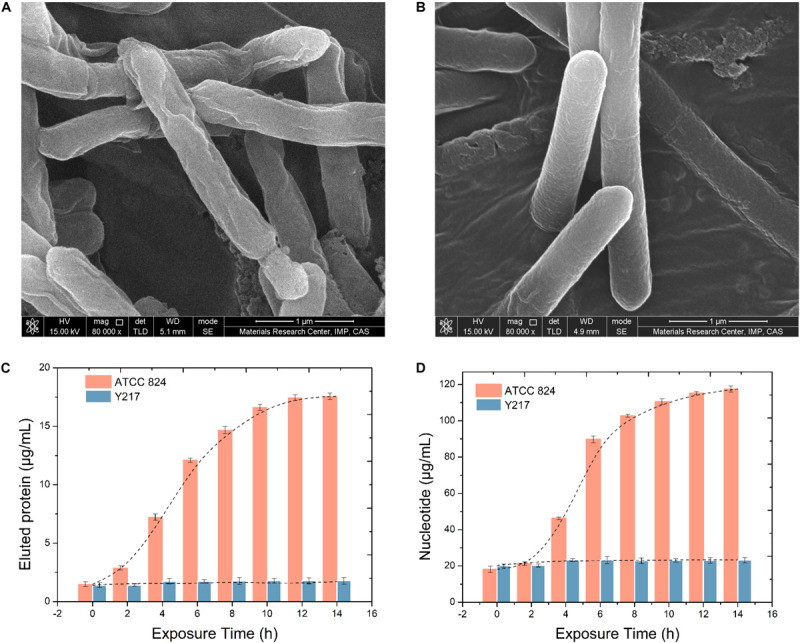
SEM images of ATCC 824 **(A)** and Y217 **(B)** cultivated in a butanol medium (15 g/L). Intracellular protein penetration **(C)** and nucleotide diffusion **(D)** of *C. acetobutylicum* under 15 g/L butanol stress. The leakage trend of ATCC 824 is S-shaped.

Figures 4A,B show the changes of the cell membrane surface produced by the two strains under the stimulation of 15 g/L butanol. It can be seen from the comparison that 15 g/L butanol caused a certain degree of damage to the surface of ATCC 824 cells, and the surface was wrinkled or even damaged, while Y217 maintained a complete and full surface morphology. Results of intracellular protein penetration and nucleotide diffusion detection of *C. acetobutylicum* ATCC 824 and Y217 under butanol stimulation are shown in Figure 4. It can be seen from Figure 4C that the protein penetration of ATCC 824 increases with extension of exposure time, and the osmolality gradually stabilizes after 10 h. Additionally, Figure 4D shows that the diffusion rate of ATCC 824 stagnated during 0–2 h and reached the logarithmic period of nucleotide diffusion from 4 to 8 h. It remained stable after 8 h, and the overall change trend was S-shaped. During the whole exposure time of Y217, no obvious protein penetration and nucleotide diffusion were observed.

### Butanol Tolerance Was Verified by RSM

In this experiment three factors, including butanol (*X*_1_), temperature (*X*_2_), and initial pH (*X*_3_), were used as independent variables, and the butanol production (*Y*) was used as the response value. The response surface analysis plan was designed to include 12 response surface analysis test points and five center points per block. The factors and levels of response surface analysis are shown in Table 1, and the test design and test results are shown in Table 2. Using Design Expert software, the response value and three factors are regression-fitted, and the ternary quadratic regression equation of Y to *X*_1_, *X*_2_, *X*_3_ is:

**TABLE 2:** Design and results of response surface experiments.

**Run No.**	***X*_1_:Butanol(g/L)**		***X*_2_:Temperature(°C)**		***X*_3_:pH**		**Y:Butanol(g/L)**	
	**Coded**	**Actual**	**Coded**	**Actual**	**Coded**	**Actual**	**Experimental**	**Predicted**
1	−1	6.00	−1	34.00	0	6.80	7.22	7.25
2	−1	6.00	0	36.50	−1	6.20	7.28	7.38
3	0	8.00	−1	34.00	−1	6.20	7.22	7.09
4	0	8.00	−1	34.00	1	7.40	7.92	8.00
5	0	8.00	0	36.50	0	6.80	8.48	8.46
6	−1	6.00	0	36.50	+1	7.40	7.94	7.84
7	0	8.00	0	36.50	0	6.80	8.46	8.46
8	0	8.00	0	36.50	0	6.80	8.48	8.46
9	+1	10.00	+1	39.00	0	6.80	7.06	7.03
10	0	8.00	0	36.50	0	6.80	8.32	8.46
11	+1	10.00	0	36.50	−1	6.20	7.04	7.15
12	−1	6.00	+1	39.00	0	6.80	7.50	7.47
13	0	8.00	+1	39.00	+1	7.40	7.42	7.56
14	0	8.00	0	36.50	0	6.80	8.54	8.46
15	+1	10.00	−1	34.00	0	6.80	7.12	7.15
16	0	8.00	+1	39.00	−1	6.20	7.70	7.63
17	+1	10.00	0	36.50	+1	7.40	7.64	7.54

$$Y=8.46-0.14\,X_{1}+0.025\,X_{2}+0.21\,X_{3}-0.085\,X_{1}\,X_{2}$$

$$-0.015\,X_{1}\,X_{3}-0.25\,X_{2}\,X_{3}-0.66\,X_{1}^{2}-0.57\,X_{2}^{2}-0.32\,X_{3}^{2}$$

The determination coefficient of the regression model *R*^2^ = 0.9752 indicates that 97.52% change in response value comes from the selected variable, and only 2.48% total variation exists. R^2^ (adjusted) = 0.9433, which is close to the correlation coefficient (R^2^) and is greater than 0.9, indicating that the test is highly reliable. Analysis of ANOVA (Table 3) of the regression model shows that *p*-value (Prob > F) is less than 0.0001, which indicates that model terms are significant. The lack of fit is not significant, indicating that the unknown error factors have little interference with the experimental results which indicate that the model fits well. Among the three factors, *X_1_, X_3_, X_2_ X_3_, X_1_^2^, X_2_^2^, and X_3_^2^* all have significant effects. Comparing the *F* values, shows that the order of influence on butanol production is: (*X*_3_) pH>*(X_1_*) Butanol>(*X*_2_) Temperature.

**TABLE 3:** ANOVA for response surface quadratic model.

**Source**	**Sum of Squares**	**Degree of freedom**	**Mean square**	***F* value**	***P* value Prob > *F***
Model	4.80	9	0.53	30.56	< 0.0001**
*X*_1_-Butanol	0.15	1	0.15	8.36	0.0233*
*X*_2_-Temperature	5.000E-003	1	5.000E-003	0.29	0.6090
*X*_3_-pH	0.35	1	0.35	20.22	0.0028**
*X*_1_*X*_2_	0.029	1	0.029	1.66	0.2390
*X*_1_*X*_3_	9.000E-004	1	9.000E-004	0.052	0.8268
*X*_2_*X*_3_	0.24	1	0.24	13.76	0.0076**
*X*_1_^2^	1.84	1	1.84	105.29	< 0.0001**
*X*_2_^2^	1.37	1	1.37	78.55	< 0.0001**
*X*_3_^2^	0.43	1	0.43	24.79	0.0016**
Residual	0.12	7	0.017		
Lack of Fit	0.095	3	0.032	4.76	0.0829
Pure Error	0.027	4	6.680E-003		
Cor Total	4.92	16			
R-Squared	0.9752				
Adj R-Squared	0.9433				

“**” means that the difference is extremely significant (P < 0.01); “*” means that the difference is significant (P < 0.05).

**FIGURE 5:**
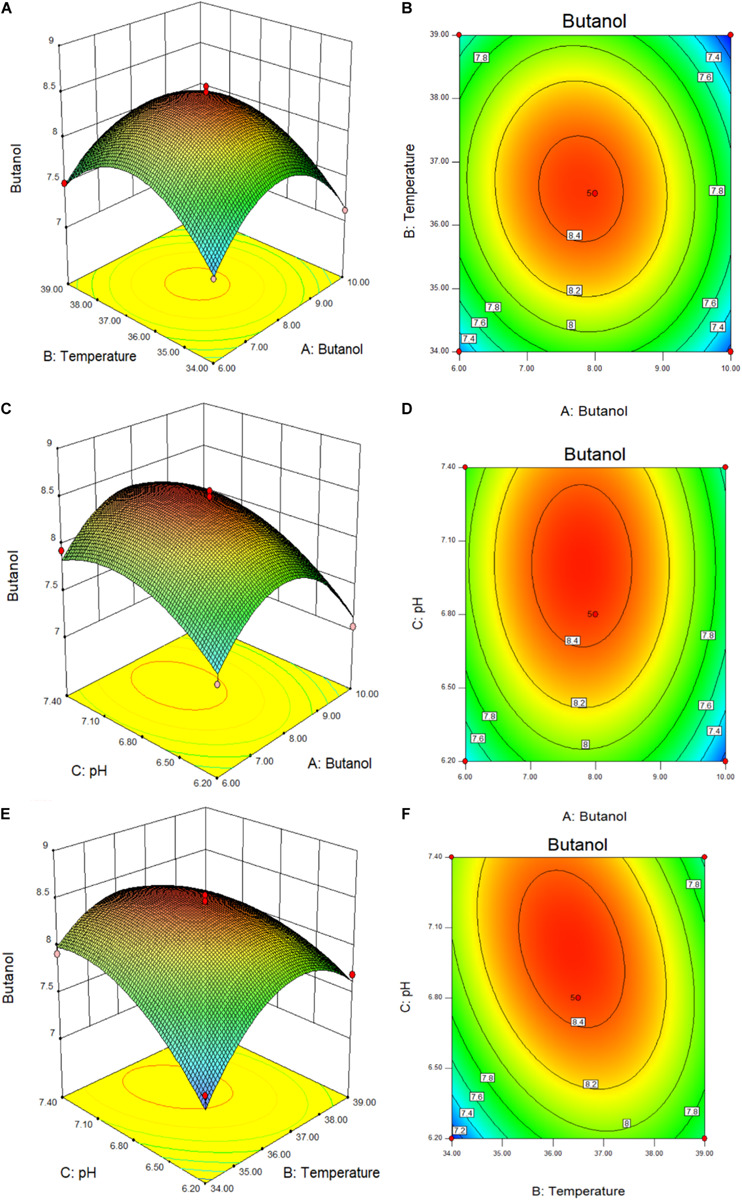
3D surfaces **(A–C)** and contour plots **(D–F)** of butanol produced by *C. acetobutylicum* mutant (Y217), showing interactions among three factors (exogenous butanol, temperature, and pH).

The response surfaces and contours of the interaction between the added butanol and temperature, the added butanol and pH, and the temperature and pH, were drawn by the Design-Expert 8.0.6 software, and the results were shown in Figures 5A–F. Tables 2, 3 and Figure 5, combined with the regression equation, indicate that the best conditions for the mutant to produce butanol when exogenous butanol is added are as follows: exogenous butanol, 7.79 g/L; temperature, 36.39°C; and initial pH, = 7.01. Under these fermentation conditions, the butanol production theoretical value is 8.49 g/L. To verify the practicability and accuracy of the model, the fermentation conditions were modified to add 7.8 g/L butanol exogenously, the fermentation temperature was 36.4°C, and initial pH = 7. Through the verification test, the final experimental result was 8.35 g/L. The output of butanol under optimal conditions is basically close to the theoretical value, indicating that the parameters of this model are reliable.

## Discussion

Carbon ion beam irradiation has a high mutation rate, and is an effective mutation-production method that has been widely used in industrial microbial breeding. Therefore, carbon ion beam irradiation can be an effective means to induce significant changes in the physiological characteristics of microorganisms. The mutant strain of baker’s yeast, with a high yield of β-glucan and screened by carbon ion beam irradiation, was optimized under laboratory fermentation conditions, and its yield was 1.73 times higher than that of the original strain YS-00 (Li et al., 2017). Although carbon ion beam irradiation has been universally used in plant and microorganism breeding, it has not been widely reported in the breeding of *C. acetobutylicum*. In this study, we found that carbon ion beam irradiation can boost the butanol production characteristics of *C. acetobutylicum*. Due to the lack of reference mutagenesis conditions, a series of carbon ion irradiation doses was applied according to the previous reports on the use of carbon ion irradiation for the breeding of microorganisms (Ma et al., 2013). The results revealed that an increase in irradiation doses enhances the mortality rate from 15 to 86% (Figure 1A). According to our experience in mutagenesis breeding, high mutation rates often accompany high mortality (Ma et al., 2013). Thus, nearly 2000 clones, with a mortality rate of at least 50%, were selected for further mutation screening under irradiation doses of 90, 120, 150, and 200 Gy. The results revealed that carbon ion beam irradiation has obvious mutation effects on *C. acetobutylicum*. With an increase in irradiation dose, the lethality of *C. acetobutylicum* irradiation was enhanced, demonstrating that *C. acetobutylicum* is sensitive to high linear energy transfer (LET) carbon ion beam irradiation.

Based on the study of the fermentation kinetic curves of the five mutant strains, we aimed to select the one with the best performance as the research object for the follow-up experiment. Results showed that the butanol yield of Y007 and Y217 both exceeded 13.50 g/L, and that of Y007 reached 13.69 g/L. The two strains with the highest total solvent yield were Y217 and Y218, and Y217 was 19.86 g/L, indicating the fermentation performance of Y217 was excellent. Although the butanol yield of Y009 only reached 13.30 g/L, the total fermentation time of Y009 was 6 h shorter than other strains, and the productivity was 0.20 g/L/h, reaching the high production rate reported in the literature. The butanol yield of the Y207 strain was the lowest of those five mutants (11.40 g/L), but it reached the highest butanol concentration at 66 h, indicating that it shortened the fermentation time to some extent. The final concentration of acid in the fermentation medium of the five mutants was lower than that of the wild-type strain, indicating that the conversion rate of mutants increased during the acidogenesis-solventogenesis transformation period. Many studies have explored both external and internal factors that contribute to the transition from acid to solvent (Heipieper et al., 2007; Yang and Zhao, 2013). External factors include extracellular pH, extracellular undissociated acid concentration, nutritional factors, and temperature. Intracellular factors include intracellular pH, intracellular NAD(P), intracellular ATP/ADP, intracellular butyryl CoA, and butyryl phosphate concentration. Extracellular pH is considered the key factor determining the effect of fermentation to produce solvents. When the extracellular pH is high the bacteria mainly produce acid. When the extracellular pH is low the bacteria tend to produce solvents (Guo et al., 2013; Yang and Zhao, 2013). However, the pH scope for promoting bacterial-producing solvents is different for different strains and fermentation conditions (Yang et al., 1992; Kilonzo et al., 2009). The optimum pH range for solvent production of *C. acetobutylicum* ATCC 824 was 5.5–4.3. The experiment result shows that the acid production of Y007 and Y217 in acidogenesis was high, and the extracellular pH was 4.54 and 4.50, respectively, which may promote the occurrence of an acidogenesis-solventogenesis transformation period, during which acetic acid and butyric acid can be transformed to acetone and butanol in solventogenesis. The increase of pH in the fermentation broth reduced the toxicity of acid to cells, and thus increased the butanol yield. As shown in Figure 2F, strain Y218 had the highest acid yield compared with other strains in the acid production stage, but its butanol yield and total solvent yield were not suitable, and its final acid concentration was higher than that of Y007 and Y217. It is evident that the fermentation phenotypes of the five mutants were related to the complex changes during acidogenesis, solventogenesis, and acidogenesis-solventogenesis transformation stages. So far, there is no consensus on the regulation of the transition from the acid-producing stage to the solvent-producing stage (Heipieper et al., 2007). This complex physiological change may not be caused by a single factor, and there may be a mechanism of multifactor coordinated comprehensive regulation. These five mutants were mutated in different directions, and eventually increased the yield of butanol. They were independent strains that were different from each other. Based on the consideration of butanol, total solvent yield, and final acid quantity, we finally chose Y217 for the determination of genetic stability. Its growth (DCW), acetone, butanol, and ethanol yields were very stable within 12 generations. Results showed that carbon ion beam radiation mutagenesis, as an effective breeding method, could be successfully applied to the breeding of *C. acetobutylicum* strains with high yield. Jiang et al. (2018) used CGM medium with 60 g/L glucose to determine the solvent production capacity of the wild-type strain and mutant with heavy ions beam irradiation, and found that the butanol concentration of the irradiated strain was 8.096 g/L for 60 h, which was 1.959 g/L higher than the control. In this study, Y217 could stably produce butanol 13.67 g/L with 70 g/L corn powder, which was 3.9 g/L higher than the control (9.77 g/L).

In the process of microbial fermentation, whether the target product can be transferred to the extracellular optimally is very important. The growth and metabolism of microorganisms are directly related to the selective permeability of the membrane to compound in and out (Yang et al., 2015; Guan et al., 2018). The permeability of the cell membrane is changed, allowing intracellular metabolites to leak out of the cell quickly, thereby preventing the accumulation of metabolites in the cell and releasing the feedback regulation effect of the end product, and thus increasing the fermentation product (Wei et al., 2006; Hao, 2007). However, changes in cell membrane permeability are also beneficial for nutrients entering the cell, and can promote cell metabolism (Hao, 2007). Studies have also been conducted on the application of ultrasound technology to enhance cell membrane permeability in the butanol fermentation process, and promising results have been achieved. When the culture was sonicated at 0 h for 15 min, butanol and total solvent production were 30.2 and 22.8% higher than those of the control group, respectively (Qureshi and Maddox, 2005; Nitayavardhana et al., 2010). Figure 4 shows that under butanol stimulation cell integrity of Y217 remains basically stable. After 4 h of butanol stimulation there was a very slight increase in permeability, and then a relatively balanced cell membrane permeability was maintained, indicating that within 4 h the cell membrane was in the delayed period of butanol stimulation. It is reasonable to assume that the cell membrane was responding to butanol stimulation during this period. Four hours later a moderate increase in membrane permeability increased the extent to which the Y217 strain transported the intracellular butanol product to the outside of the cell to reduce product inhibition, and thereby increased butanol production. Without lethal effect, 15 g/L butanol increased membrane permeability of Y217 and had a positive effect on butanol production. In contrast, 15 g/L butanol is fatal to ATCC 824, resulting in excessive intracellular extravasation and ultimately, loss of cell fermentability or death. Transmembrane transport and phosphorylation of glucose and other substances also depend on favorable membrane permeability. A key factor in determining whether nutrients can be used by microorganisms is if these nutrients can enter microbial cells. Only after the nutrients enter the cell can they be decomposed and utilized by the metabolic system, which in turn allows the normal growth and metabolism. Phosphoenolpyruvate-carbohydrate phosphotransferase system (PTS) is the main way for carbohydrates to enter the cells of *C. acetobutylicum*. Sugars such as glucose, fructose, and mannose are phosphorylated, mainly by the PTS system located on and in the membrane, and then enter the cell in the form of phosphorylated sugar (Lengeler, 1996). The diffusion of macromolecules under the action of butanol by Y217 and ATCC 824 shows that the same butanol concentration has a completely different effect on different strains. The toxic effects of solvents on bacteria mainly results in damage to the cell membrane (Weber and de Bont, 1996). Some prior studies indicate that engineering strategies regulating the membrane composition can be a potential target in the future for tuning membrane permeability to increase the stress tolerance of industrial strains. Thus, we believe that Y217 may be able to adapt to the solvent and improve the tolerance by changing the lipid composition of the membrane structure and membrane protein functions. Thus, Y217 may resist cell membrane permeability damage caused by butanol stress, maintain the proton gradient and cell electron potential, and avoid cell inactivation or death caused by the large leakage of intracellular proteins and nucleic acids due to the increased permeability (Ezeji et al., 2010; Isar and Rangaswamy, 2012). RSM analysis was used to verify the ability of Y217 to withstand high concentrations of butanol stress.

The 3D surface plots and contour plots of RSM are shown in the Figure 5. The 3D surface plot depicts the interaction between the other two variables in the experimental range when one of the variables remains constant at zero (Lin et al., 2011; Al-Shorgani et al., 2015). The slope of the surface reflects the strength of the interaction between the two experimental factors. Similarly, different shapes of contour plots indicate different interactions between variables. When there is perfect interaction between variables an elliptical profile is obtained. The response surface curve shows the influence of the interaction between various factors on the fermentation butanol (Sun et al., 2020). The steeper the response surface curve, the stronger is the interaction. If the contour line is elliptic, it indicates that the interaction of this factor has a great influence on the fermentation butanol. Figures 5A,B show that the contour line is oval, indicating that the added butanol concentration and temperature have a strong interaction. Furthermore, Figures 5C,D show that the gradient of added butanol concentration and pH is very steep, indicating that they have a great influence on the butanol production. Moreover, the contour line along the butanol addition axis is denser than the pH, indicating that when the two interact the external butanol addition has a greater influence on butanol fermentation than pH. From Figures 5E,F, it can be seen that the contour line is elliptic and that the response surface is extremely steep, indicating that temperature and pH interact, and the effect on the fermentation butanol differed significantly. RSM is commonly used to optimize fermentation process conditions to increase butanol and solvent production. Long et al. (2013) used fresh plantain as raw material to produce butanol with *C. acetobutylicum* CICC 8012. The central composite design was used to optimize the fermentation conditions and establish a mathematical model with butanol yield as the response value. Under optimized conditions butanol fermentation yield reached 12.73 g/L. Figueroa-Torres et al. (2020) evaluated the yield of ABE fermentation solvents under different initial medium concentrations of acetic acid and butyric acid by response surface analysis. In this study, RSM was used to verify the butanol tolerance of Y217, which can grow normally and ferment to produce butanol 7.04–8.54 g/L in 6–10 g/L added-butanol medium. The optimal conditions obtained by model optimization are: in 7.8 g/L butanol medium, 8.35 g/L butanol can be produced, and the final butanol concentration at the end of fermentation can reach 16.15 g/L, indicating that Y217 is also a high-tolerance butanol strain with good fermentation performance. To investigate the relevant mechanisms of butanol tolerance of strains, we will conduct extensive research on the cellular and genetic levels in the future.

## Conclusion

In this study, we demonstrated the excellent butanol production and tolerance of mutant Y217 selected by carbon ion beam irradiation. The maintenance of its cell membrane surface structure and permeability under butanol stress, in particular, was demonstrated. The organic solvent environmental stress can cause changes in the structure of the protein or lipid molecules that make up the membrane structure such that while the fluidity of the cell membrane is affected, the permeability of the plasma membrane is often increased, and the selective permeability is reduced. External leakage causes changes in the structure and function of the cell membrane. Therefore, the cell membrane of the mutant Y217 not only forms a permeability barrier that regulates the entry channel between the cell and the external environment, but also has special significance for intracellular energy conduction, and different cell membrane structures directly affect the solvent resistance of the mutant. Therefore, further studies should explore the physiological and biochemical characteristics of cell membranes; further, the relevant metabolic mechanisms can be understood using omics to provide a theoretical basis for future breeding and construction of high-quality production bacteria.

## Data Availability Statement

The original contributions presented in the study are included in the article/Supplementary Material, further inquiries can be directed to the corresponding author.

## Author Contributions

DL and XZ coordinated and supervised the project. YG designed the experiments and wrote the manuscript. YG and CL analyzed the data. YG, MZ, and XG performed the experiments. MZ, DL, and WL corrected the manuscript. All authors read and approved the final manuscript.

## Conflict of Interest

The authors declare that the research was conducted in the absence of any commercial or financial relationships that could be construed as a potential conflict of interest.
