# Supplementary material for: Effects of Carbon Ion Beam Irradiation on Butanol Tolerance and Production of Clostridium acetobutylicum
Source: Front Microbiol. 2020 Dec 18;11:602774. doi: 10.3389/fmicb.2020.602774 (PMC7775398; doi:10.3389/fmicb.2020.602774)
Supplement: Supplementary file 1 [file Data_Sheet_1.PDF]

Supplementary material

Effects of carbon ion beam irradiation on butanol tolerance and production of *Clostridium acetobutylicum*

Yue Gao[1,2], Miaomiao Zhang[1,2,3], Xiang Zhou[1,2], Xiaopeng Guo[1,2], Cairong Lei[1,2], Wenjian Li[1,2,3], Dong Lu[1,2,3]*

[1]Institute of Modern Physics, Chinese Academy of Sciences, Lanzhou, China

[2] University of Chinese Academy of Sciences, Chinese Academy of Sciences, Beijing, China

[3]Gansu Key Laboratory of Microbial Resources Exploitation and Application, Lanzhou, China.

*** Correspondence: Dong Lu**
Corresponding Author
LD@impcas.ac.cn

To determine the phenotype of mutant strain Y217 with its genotype and to guide the metabolic engineering of *C. acetobutylicum*, we conducted whole-genome resequencing. The raw sequence data of mutant Y217 have been deposited in the Genome Sequence Archive in National Genomics Data Center, Beijing Institute of Genomics (China National Center for Bioinformation), Chinese Academy of Sciences, under accession number **CRA003426** that are publicly accessible at https://bigd.big.ac.cn/gsa.

Using SnpEff to annotate the distribution of single nucleotide polymorphism (SNPs) and small InDel mutations in various regions (intergenic regions and gene coding regions, etc.) of the genome, and the position of the mutation site on the reference genome or the region where the mutation occurred in the genome was obtained. In the GO annotation classification of the mutant strain Y217 (Figure 1), it can be seen that the different gene functions are more annotated to metabolic process, cellular process, catalytic activity, which also reflects the metabolic and physiological preference of the

mutant strain Y217. Through the screening and confirmation of mutant genes, we finally paid attention to six mutation sites that may be related to the excellent phenotype of Y217, including base substitution and small fragment insertion (shown in List 1). Mutant genes include the EIID protein-encoding gene of the phosphoenolpyruvate-carbohydrate phosphotransferase system (PTS^{ManII}) transport system related to sugar transport and two proteins related to ABC transporter, which may be involved in regulating butanol metabolism of Y217 in the process of affecting substance transport. The genes that may be associated with butanol tolerance are CA_C0080 and CAC_3088, which encode components of the bacterial two-component regulation system (TCSs), respectively. In addition, there is a predicted membrane protein, and the related mechanism can be further studied (Table 1).

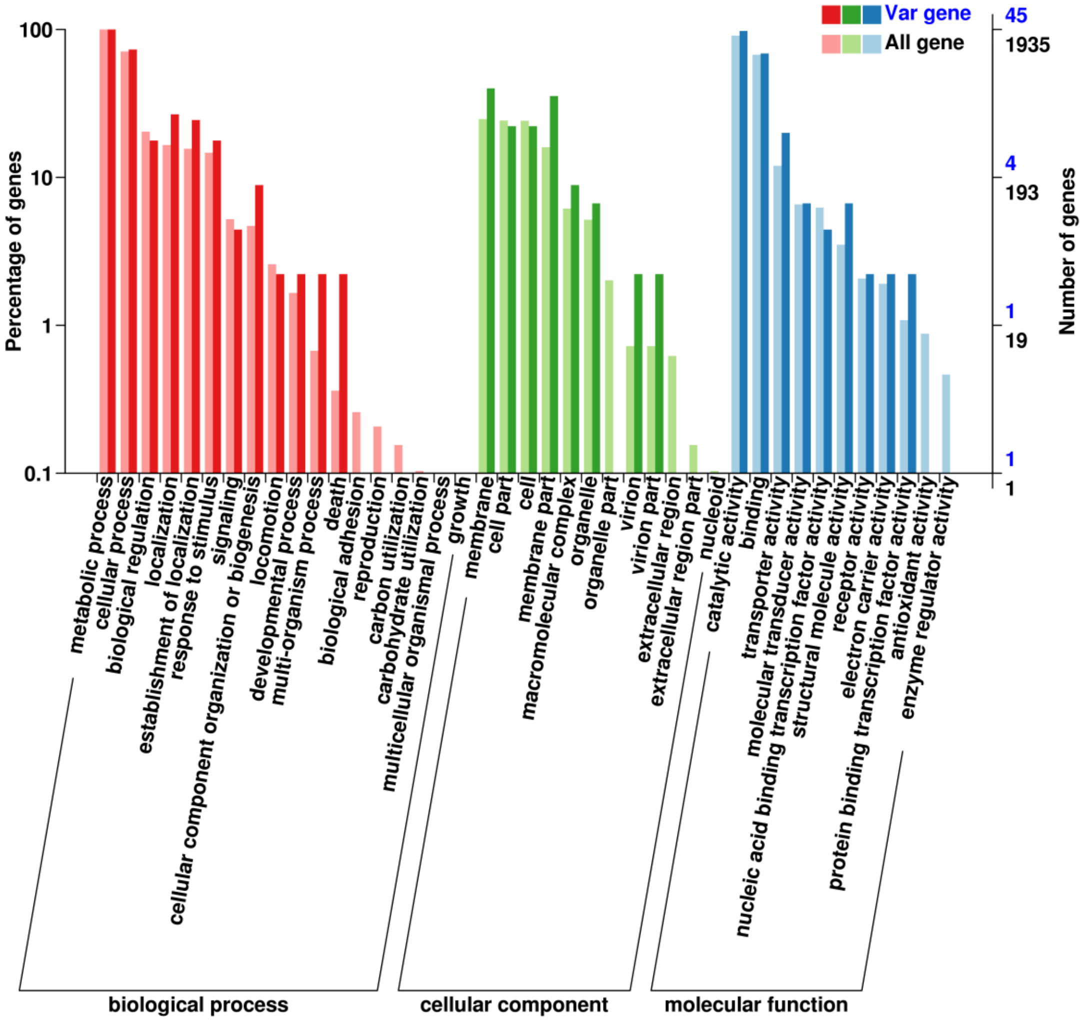

Figure 1 Mutant strain Y217 variant gene GO annotation classification map. The x-coordinate is the contents of each GO classification, the left coordinate is the percentage of the number of genes, and the right coordinate is the number of genes.

List 1 of mutant sites

1. **NC_001988.2 position: 71766 Gene position: 389**

ATCC 824: 381 agcagcttca cttgctatga gtggtaacat
Y217: 381 agcagcttta cttgctatga gtggtaacat

2. **NC_003030.1 position: 88760 Gene position: 1144**

ATCC 824: 1141 aatgaggaaa ttccaccaat atacaagatt
Y217: 1141 aattaggaaa ttccaccaat atacaagatt

3. **NC_003030.1 position: 44134 Gene position: 1003**

ATCC 824: 991 aaaatccgta tcagagttta attttttaaa
Y217: 991 aaaatccgta tctgagttta attttttaaa

4. **NC_003030.1 position: 3242782 Gene position: 1281**

ATCC 824: 1261 ttaatagaaa gtgaactttt tggctatgaa
Y217: 1261 ttaatagaaa gtgaactttt gggctatgaa

5. **NC_003030.1 position: 3478515 Gene position: 1272**

ATCC 824: 1261 ccttcctact gt ——— tcctactg cgttaactgt tcctaatact
Y217: 1261 ccttcctact gtctt…agctcctactg cgttaactgt tcctaatact

6. **NC_003030.1 position: 1036382 Gene position: 354**

ATCC 824: 331 ttcacgcagt acatccttaa tttg acctga
Y217: 331 ttcacgcagt acatccttaa tttgtacctga

Table1 Details of mutation locations and types of mutant genes related to possible butanol metabolism and tolerance function in Y217.

Mutant types	Chromosome position	Gene Position	Effect	Base change	Comments for gene product	Gene ID
SNPs	NC_001988.2 71766	389	Non-synonymous coding	tCa/tTa	PTS mannose/fructose/sorbose transporter family subunit IID	CA_P 0068
	NC_003030.1 44134	1003	Non-synonymous coding	Aga/Tga	ABC1 family protein kinase	CA_C 0033
	NC_003030.1 88760	1144	Non-synonymous coding	Gag/Tag	Sensor histidine kinase	CA_C 0080
	NC_003030.1 3242782	1281	Non-synonymous coding	ttT/ttG	NtrC family transcriptional regulator, ATPase domain fused to two PAS domains	CA_C 3088
	NC_003030.1 3304782	209	Upstream	--	Acetolactate synthase large subunit	CA_C 3169
InDels	NC_003030.1 1036382	354	Frame shift Codon insertion	ttG/ttGT	ABC transporter ATPase, ATP-dependent Clp protease ATP-binding subunit	CA_C 0904
	NC_003030.1 3478515	1272	Codon change Codon insertion	tgttcc/tgtCTT(~210)AGCtcc	Predicted membrane protein	CA_C 3309
